# Popular Nutrition-Related Mobile Apps: A Feature Assessment

**DOI:** 10.2196/mhealth.5846

**Published:** 2016-08-01

**Authors:** Rodrigo Zenun Franco, Rosalind Fallaize, Julie A Lovegrove, Faustina Hwang

**Affiliations:** ^1^ School of Systems Engineering University of Reading Reading United Kingdom; ^2^ Hugh Sinclair Unit of Human Nutrition and Institute for Cardiovascular and Metabolic Research Department of Food and Nutritional Sciences University of Reading Reading United Kingdom

**Keywords:** nutrition apps, diet apps, food diary, nutritional assessment, mHealth, eHealth, mobile phone, mobile technology

## Abstract

**Background:**

A key challenge in human nutrition is the assessment of usual food intake. This is of particular interest given recent proposals of eHealth personalized interventions. The adoption of mobile phones has created an opportunity for assessing and improving nutrient intake as they can be used for digitalizing dietary assessments and providing feedback. In the last few years, hundreds of nutrition-related mobile apps have been launched and installed by millions of users.

**Objective:**

This study aims to analyze the main features of the most popular nutrition apps and to compare their strategies and technologies for dietary assessment and user feedback.

**Methods:**

Apps were selected from the two largest online stores of the most popular mobile operating systems—the Google Play Store for Android and the iTunes App Store for iOS—based on popularity as measured by the number of installs and reviews. The keywords used in the search were as follows: calorie(s), diet, diet tracker, dietician, dietitian, eating, fit, fitness, food, food diary, food tracker, health, lose weight, nutrition, nutritionist, weight, weight loss, weight management, weight watcher, and ww calculator. The inclusion criteria were as follows: English language, minimum number of installs (1 million for Google Play Store) or reviews (7500 for iTunes App Store), relation to nutrition (ie, diet monitoring or recommendation), and independence from any device (eg, wearable) or subscription.

**Results:**

A total of 13 apps were classified as popular for inclusion in the analysis. Nine apps offered prospective recording of food intake using a food diary feature. Food selection was available via text search or barcode scanner technologies. Portion size selection was only textual (ie, without images or icons). All nine of these apps were also capable of collecting physical activity (PA) information using self-report, the global positioning system (GPS), or wearable integrations. Their outputs focused predominantly on energy balance between dietary intake and PA. None of these nine apps offered features directly related to diet plans and motivational coaching. In contrast, the remaining four of the 13 apps focused on these opportunities, but without food diaries. One app—FatSecret—also had an innovative feature for connecting users with health professionals, and another—S Health—provided a nutrient balance score.

**Conclusions:**

The high number of installs indicates that there is a clear interest and opportunity for diet monitoring and recommendation using mobile apps. All the apps collecting dietary intake used the same nutrition assessment method (ie, food diary record) and technologies for data input (ie, text search and barcode scanner). Emerging technologies, such as image recognition, natural language processing, and artificial intelligence, were not identified. None of the apps had a decision engine capable of providing personalized diet advice.

## Introduction

Noncommunicable diseases such as diabetes and cardiovascular diseases account for almost two-thirds of deaths globally. The general recommendations for addressing this epidemic are related to lifestyle changes, mainly encouraging healthy diets, physical activity (PA), and the reduction of tobacco use and alcohol consumption [1].

Valid dietary intake recording is key for nutritional intervention. The methods used for collecting food intake data can be classified in a number of ways. Based on the time of the collection, the retrospective methods, such as the 24-hour food recall and the food frequency questionnaire (FFQ), require memory for recollection of foods eaten. In contrast, the prospective methods require diet reporting as the consumption occurs, acting as food diaries. In clinical nutrition, prospective methods are usually applied between 4 and 7 days. It is also possible to classify the methods as quantitative daily consumption or food frequencies. The first group focuses on recording the detailed food consumption as accurately as possible, typically for a couple of days. The latter assesses typical consumption patterns over longer periods [2]. These methods have been delivered traditionally using a paper-and-pen format, but there is a burden associated with this system for both the patients and health professionals. The digitalization of food diaries saves time and resources and is preferred by patients [3].

With the proliferation of mobile phones and tablets, there has been a rise in the number of software apps aimed at improving nutrition and physical fitness. The simple digitalization of input data is important and useful, but these devices have built-in capabilities that can increase the accuracy of data collection and decrease the time burden of the process and possible biases [4]. The most common example is the use of the global positioning system (GPS) for measuring PA [5]. Cameras can be used for image recognition in order to recognize foods and estimate portion sizes [6,7]. In relation to the use of technology to encourage behavior changes, there are studies and available diet apps that combine health behavior theories and persuasive technology [8]. This topic is particularly important because one of the main goals of nutrition intervention is to modify unhealthy habits.

Due to the large number of nutrition-related apps, it is difficult to understand what these apps are offering and how the apps compare with each other. This study aims to review the main features and technologies used by popular nutrition-related apps available in the online market and to analyze their use of emerging technologies in the field of online nutrition assessment and intervention. This review will be beneficial for industry, academia, and health professionals who are interested in taking advantage of the benefits of technology in nutrition assessment and intervention.

## Methods

During the publication of a mobile app, a developer specifies in which stores—usually divided by countries—the app will be available. They also specify what device requirements (eg, versions of the operating system and mobile phone or tablet) are necessary in order to install the app. Searching for apps from a specific device in a particular country can alter the apps that appear available to the user. In order to mitigate this, the initial search was conducted on a desktop personal computer (PC) not logged into any particular user account, but located in the United Kingdom. Searches were conducted in November 2015.

For the Google Play Store, the initial search was executed using the Google Chrome browser in an incognito window (ie, private mode), logged off from the Google account, using the following keywords: calorie(s), diet, diet tracker, dietician, dietitian, eating, fit, fitness, food, food diary, food tracker, health, lose weight, nutrition, nutritionist, weight, weight loss, weight management, weight watcher, and ww calculator. An initial list of popular apps, ordered by number of installs and reviews, was created. For the iTunes App Store, the initial search was performed via iTunes—software provided by Apple—logged off from any user account. The apps were ordered by number of reviews because the App Store does not list the number of installs. The user rating was used as an exclusion criterion. The rating range is between 0 and 5 and represents the user satisfaction with the app, with 5 being the most satisfied. Apps were excluded if ratings were below 3, in order to avoid considering apps that were downloaded by many users but may not be in use (eg, because they were not working properly or did not deliver what was advertised in the store). Apps which only monitored weight or PA, such as Google Fit, or that only provided recipes were also not considered. After the creation of an initial list of apps, user accounts linked with a UK address and credit card were used to install the apps and verify the apps against the inclusion criteria.

Once the apps were installed, their features were reviewed from both nutritional and technological perspectives. From the nutritional perspective, features in the following categories were considered: dietary intake, phenotype, physical activity, and others. The technological perspective analyzed what technologies were being used in order to compare with emerging technologies in the field of human nutrition assessment and intervention. The functionalities were analyzed in two main groups: input and output features. Features that required data from the user (eg, weight and height) were considered as input features, while the results shown to the user were termed output features.

## Results

### App Selection

In the Google Play Store, it is not possible to sort the results by number of installs. It has an internal algorithm that classifies the relevance of the apps and presents them in a list. For this reason, it was necessary to open the first 20 results by keyword to get the number of installs in order to mitigate the risk of missing an app with a high number of installs. The app list created in this process was ordered by number of installs and a total of 21 apps with greater than 500,000 installs were identified (see Table 1). To further reduce the number of apps for inclusion—for practical reasons and readability of results—apps with less than 1 million installs were excluded.

**Table 1:** Popular (>500,000 installs) nutrition-related apps available in the UK Google Play Store.

App name^a^	Abbreviation	Installs (range), n	Reviews, n	Rating (0-5)
S Health-Fitness Diet Tracker	SH	100m-500m	33,619	3.7
Calorie Counter-MyFitnessPal	MFP	10m-50m	1,140,897	4.6
Calorie Counter by FatSecret	FS	10m-50m	178,438	4.3
Noom Coach: Weight Loss Plan	NC	10m-50m	161,237	4.3
My Diet Coach-Weight Loss^b^	MDC	5m-10m	102,318	4.3
Lose it! by FitNow Inc	LI	5m-10m	45,391	4.4
Weight Watchers Mobile^c^	WW	1m-5m	66,897	3.9
Lose Weight Without Dieting	LW	1m-5m	56,617	4.6
Lifesum-The Health Movement	LS	1m-5m	46,856	4.2
Diet Point-Weight Loss by Diet Point^d^	DP	1m-5m	28,906	4.2
My Diet Diary Calorie Counter	MDD	1m-5m	17,711	4.1
Effective Weight Loss Guide^d^	EWL	1m-5m	16,156	4.1
Diet Assistant-Weight Loss^d^	DA	1m-5m	10,722	3.9
Calorie Counter by Calorie Count	CC	1m-5m	7529	4.0
MyNetDiary Calorie Counter PRO^e^	N/A^f^	500,000-1m	10,405	4.4
Weight Watchers Mobile UK^e^	N/A	500,000-1m	9896	3.7
Calorie Counter & Diet Tracker^e^	N/A	500,000-1m	9306	4.3
WWDiary by Canofsleep^e^	N/A	500,000-1m	8564	4.6
Calorie, Carb & Fat Counter^e^	N/A	500,000-1m	7923	4.3
Diet Plan-Weight loss 7 days^e^	N/A	500,000-1m	5013	3.8
Calculator & Tracker for WWPP^e^	N/A	500,000-1m	1898	3.8

^a^Results from November 2015.

^b^My Diet Coach provides some diet recommendations in the free version. The food diary is available only in the *Pro* version, which was not considered one of the most popular apps in this study.

^c^This app was later excluded due to subscription.

^d^Diet Point, Effective Weight Loss, and Diet Assistant are not food diaries, but they provide diet recommendations via diet plans.

^e^These apps were later excluded due to minimum threshold.

^f^N/A: not applicable.

All of the apps were in the “health & fitness” category of the store. No app was excluded by the rating criterion (ie, rating <3). However, although the Weight Watchers (WW) app is free to download, a subscription—£12.95 monthly for the online plan—was required to join the online program [9] and thus it was excluded from subsequent analysis.

The same search keywords were used in the iTunes App Store (see Table 2).

**Table 2:** Nutrition-related apps available in the UK iTunes App Store, ordered by number of reviews.

App name^a^	Abbreviation	Reviews, n	Rating (0-5)
Calorie Counter and Diet Tracker by MyFitnessPal	MFP	108,072	4+
Calorie/KJ Counter and Food Diary by MyNetDiary	N/A^b^	6484	3.5
Calorie/KJ Counter PRO by MyNetDiary	N/A	3818	4+
Lifesum-Healthier living, better eating	N/A	2952	3.5
Tap and Track-Calorie Counter	N/A	2317	3.5
Easy Weight Loss Tips, by Michael Quach^c^	N/A	2286	2.5
Calorie Counter and Diet Tracker by Calorie Count	N/A	1716	4
Calorie Counter+ by Nutratech	N/A	1501	4+
Argus-Calorie Counter and Activity Tracker	N/A	1291	4
Calorie Counter by FatSecret	N/A	1048	3.5

^a^Results from November 2015.

^b^N/A: not applicable.

^c^This app was not included in the analysis due to a rating of less than 3.

One app did not meet the rating criterion—Easy Weight Loss Tips, by Michael Quach, rating 2.5—and was, therefore, excluded. The most reviewed app— Calorie Counter and Diet Tracker by MyFitnessPal (MFP), with 108,072 reviews—had around 17 times more reviews than the second-most reviewed app, which had 6484 reviews. As the latter had fewer reviews than the least popular of the apps included from the Google Play Store—Calorie Counter by Calorie Count (CC), with 7529 reviews—only MFP was considered suitable for inclusion in the study. However, since MFP had already been included from the Google Play Store list and because an initial assessment of both the Google Play Store and iTunes App Store versions of the app did not reveal any notable differences, only the Google Play Store version was used in subsequent analysis.

### Input Features

Input features were analyzed for four categories of recording: dietary intake, phenotype, PA, and others (eg, personal reminders) (see Tables 3 and 4).

**Table 3:** Nutrition-related app input features for dietary intake and phenotype.

Feature/app		SH^a^	MFP^b^	FS^c^	NC^d^	LI^e^	LW^f^	LS^g^	MDD^h^	CC^i^	MDC^j^	DP^k^	EWL^l,m^	DA^n,o^
**Dietary intake**														
	Text search	✓	✓	✓	✓	✓	✓	✓	✓	✓	N/A^p^	N/A	N/A	N/A
	Barcode scanner		✓	✓	✓	✓		✓	✓	✓	N/A	N/A	N/A	N/A
	Serving size	✓	✓	✓	✓	✓	✓	✓	✓	✓	N/A	N/A	N/A	N/A
	Food by meal	✓	✓	✓	✓	✓	✓	✓	✓	✓	N/A	N/A	N/A	N/A
	Favorite foods	✓	✓	✓	✓	✓	✓	✓	✓	✓	N/A	N/A	N/A	N/A
	Create meal or recipe		✓			✓		✓			N/A	N/A	N/A	N/A
	Add kcal/kJ			✓		✓		✓			N/A	N/A	N/A	N/A
	Water consumption	✓	✓				✓	✓	✓	✓	✓	✓	N/A	N/A
	Water settings	✓						✓						
	Macronutrients settings							✓			N/A	N/A	N/A	N/A
	Save photo						✓			✓				
**Phenotype**														
	Current weight	✓	✓	✓	✓	✓	✓	✓	✓	✓	✓	✓	✓	✓
	Height	✓	✓	✓	✓	✓	✓	✓	✓	✓		✓	✓	✓
	Gender	✓	✓	✓	✓	✓	✓	✓	✓	✓	✓	✓	✓	✓
	Age/date of birth	✓	✓	✓		✓	✓	✓	✓	✓		✓	✓	✓
	Waist circumference		✓				✓							
	Hips circumference		✓				✓							
	Neck circumference		✓				✓							
	Target weight		✓	✓	✓	✓	✓	✓		✓	✓			✓
	Target date^q^				✓	✓		✓						
	Body type									✓				

^a^SH: S Health.

^b^MFP: MyFitnessPal.

^c^FS: FatSecret.

^d^NC: Noom Coach.

^e^LI: Lose it!.

^f^LW: Lose Weight Without Dieting.

^g^LS: Lifesum.

^h^MDD: My Diet Diary.

^i^CC: Calorie Count.

^j^MDC: My Diet Coach.

^k^DP: Diet Point.

^l^EWL: Effective Weight Loss.

^m^Weight and height for body mass index (BMI) calculation. Age and gender for calorie calculation.

^n^DA: Diet Assistant.

^o^Weight and height for BMI calculation. Age and gender for profile.

^p^N/A: not applicable. These features were assessed only in apps providing food diaries.

^q^Target date in *Lose it!* is set indirectly via the plan to lose fractions of kg per week.

**Table 4:** Nutrition-related app features for physical activity and other input features.

Feature/app		SH^a^	MFP^b^	FS^c^	NC^d^	LI^e^	LW^f^	LS^g^	MDD^h^	CC^i^	MDC^j^	DP^k^	EWL^l^	DA^m^
**Physical activity**														
	Type of PA^n^	✓	✓	✓	✓	✓	✓	✓	✓	✓				
	Native GPS^o^	✓			✓									
	Third-party GPS integration^p^		✓	✓	✓	✓		✓						
	Integration with wearables^q^	✓	✓		✓	✓		✓						
	Pedometer	✓			✓									
	Average activity level	✓	✓	✓			✓		✓	✓				✓
	Exercise goal	✓	✓		✓						✓			
**Other features**														
	Community forums		✓	✓		✓			✓	✓	✓	✓		✓
	Personal reminders	✓	✓		✓	✓	✓	✓			✓	✓		
	Challenges	✓								✓	✓			
	Health conditions								✓					
	Daily notes	✓								✓				

^a^SH: S Health.

^b^MFP: MyFitnessPal.

^c^FS: FatSecret.

^d^NC: Noom Coach.

^e^LI: Lose it!.

^f^LW: Lose Weight Without Dieting.

^g^LS: Lifesum.

^h^MDD: My Diet Diary.

^i^CC: Calorie Count.

^j^MDC: My Diet Coach.

^k^DP: Diet Point.

^l^EWL: Effective Weight Loss.

^m^DA: Diet Assistant.

^n^MDD does not calculate the energy by type of activity, but asks the user to enter the amount of calories spent in the physical activity (PA).

^o^GPS: global positioning system.

^p^MFP integrates with other apps provided by the same company. FS integrates with Google Fit.

^q^LS provides wearable integration only after upgrade to paid version.

**Table 5:** Nutrition-related app output features.

Feature/app		SH^a^	MFP^b^	FS^c^	NC^d^	LI^e^	LW^f^	LS^g^	MDD^h^	CC^i^	MDC^j^	DP^k^	EWL^l^	DA^m^
**Nutrition assessment**														
	Calculated energy (kcal)	✓	✓	✓	✓	✓	✓	✓	✓	✓	N/A^n^	✓	✓	N/A
	Macronutrients distribution (%)	✓	✓	✓		✓	✓	✓	✓	✓	N/A	N/A	N/A	N/A
	Micronutrients intake (thresholds)	✓	✓	✓		✓				✓	N/A	N/A	N/A	N/A
	Nutrition facts	✓	✓	✓	✓	✓		✓	✓	✓	N/A	N/A	N/A	N/A
	Calories by meal	✓	✓	✓	✓	✓	✓	✓	✓	✓	N/A	N/A	N/A	N/A
	Recommended water consumption	✓					✓	✓			✓	✓		
	Maximum calories to reach a target weight		✓	✓	✓	✓	✓	✓	✓	✓		✓		
	Calories of the new recipe		✓	✓		✓		✓			N/A	N/A	N/A	N/A
	Diet plan						✓					✓	✓	✓
	Shopping list											✓		
**PA^º^ and phenotype**														
	Energy by type of PA^p^	✓	✓	✓	✓	✓	✓	✓		✓				
	Weight progress	✓	✓	✓	✓	✓	✓		✓	✓	✓	✓		✓
	Circumferences monitoring		✓				✓							
	Body mass index	✓					✓					✓	✓	✓
**Other output features**														
	Forums or blogs		✓	✓		✓			✓	✓		✓		✓
	Social media sharing		✓		✓		✓			✓	✓	✓		
	Private social media		✓	✓		✓				✓				
	Sharing with professionals			✓										
	Healthy habits/ rewards	✓									✓			

^a^SH: S Health.

^b^MFP: MyFitnessPal.

^c^FS: FatSecret.

^d^NC: Noom Coach.

^e^LI: Lose it!.

^f^LW: Lose Weight Without Dieting.

^g^LS: Lifesum.

^h^MDD: My Diet Diary.

^i^CC: Calorie Count.

^j^MDC: My Diet Coach.

^k^DP: Diet Point.

^l^EWL: Effective Weight Loss.

^m^DA: Diet Assistant.

^n^N/A: not applicable. Features assessed only in apps providing food diaries.

^o^PA: physical activity.

^p^MDD does not calculate PA since it asks for the amount of calories instead of type of PA and duration.

My Diet Coach (MDC), Diet Point (DP), Effective Weight Loss (EWL), and Diet Assistant (DA) were not evaluated for some criteria because they are not food diaries; rather, they propose diet recommendations using different approaches. Food items could be selected by *text search* in all food diaries (n=9) or via *barcode scanner* in seven of them. *Serving sizes* could be selected using units (eg, grams) or household portion sizes (eg, teaspoon) according to the food item. Daily meals were fixed (eg, breakfast, lunch, dinner, and snacks) and the food input was divided by meal (ie, *food by meal*) in all food diaries (n=9). Only three apps—MFP, Lose it! (LI), and Lifesum (LS)—provided a feature for the users to create and save personal meals or recipes by combining existing food items in the apps. FatSecret (FS), LI, and LS also had a feature for adding calories— *quick add kcal* or *add kjoule* —without entering a food name. Lose Weight Without Dieting (LW) and CC had a feature for taking a picture of the meal, which can be used to remind the user about the food items for later entry. This feature is useful when the user does not have time to log the items during or just after the meal.

The most common phenotype inputs were current weight, height, gender, and age (see Table 3). Circumferences (ie, waist, hips, and neck) were found in two apps—MFP and LW—and entered optionally after the initial registration. In some apps, the user could also enter a target weight (n=9) and the target date (n=3) expected to reach this personal goal. When setting the target weight, Noom Coach (NC) limited the weight loss to a maximum of 1 kg per week. CC was the only app that asked the user to input their body type (ie, small, medium, or large).

For reporting PA (see Table 4), users could input the activity name and the duration in minutes (ie, feature *type of PA*). As most mobile phones have GPS hardware, they are able to perform location tracking. Accelerometers are also used for detecting the number of steps taken by the user (ie, *Pedometer*). Instead of performing movement tracking natively in the app (ie, feature *native GPS*), some apps (n=5) receive location information from other apps (ie, *third-party GPS integration*) or integrate with wearable devices (n=5) such as Fitbit, which measures distance using its internal hardware and software [10]. These wearable devices are acquired by the user separately and can be used independently of these nutrition-related apps. The *average activity level* refers to the self-report level of activity of the user (ie, low, moderate, or high).

Eight of the apps had internal forums, similar to blogs, where users post questions and recipes and can share information (see Table 4). Some apps offered the possibility of creating *personal reminders*, which could be used, for example, to remind users of snacks during the day. Some apps proposed diet challenges to users. For example, MDC users could log when they “fill half of the plate with vegetables.”

My Diet Diary (MDD) was the only software that required information about *health conditions*, including a specific mandatory input field about diabetes. Two apps offered the possibility of saving *daily notes*. S Health (SH) had data input features for caffeine tracking, blood glucose, and blood pressure.

### Output Features

Output features refer to the data and results presented by the app to the users. In terms of nutrition assessment and diet recommendation, food diaries had similar features in terms of feedback on calories and macronutrients (ie, protein, fat, and carbohydrates) (see Table 5).

Five apps provided information on micronutrient intake. MFP and SH provided tables with the daily micronutrient intake (eg, sodium, potassium, vitamin C, and iron) and the consumption *goal* and *left*. MFP provided some educational tips just after the food entry, for example, “this food is high in protein” and “this food has 1168 mg of sodium, your goal for today is to stay below 2300 mg.” Similar tips from other apps were more general and not based on the last food entry. Recommendations for water consumption (eg, “8 cups per day”) were given in five apps. After the selection of a food item, the user could examine the *nutrition facts* of the item in a way similar to the tables used in industrialized foods (n=8). LW offered a meal suggestion combining some food items that meet the suggested number of calories for the meal.

The apps that monitored dietary intake did not provide diet plans. In contrast, diet plans were the focus of DP, EWL, and DA. These apps suggested diet plans, divided by meals during the day. DP also suggested a related shopping list to the users. MDC followed a distinct approach providing generic diet recommendations via challenges and tips. Some examples of these general tips are “drink a flavored coffee (up to two cups a day),” “reduce your carbs consumption,” “restrain yourself, eat an apple instead,” and “eat a low fat yogurt.”

In terms of nutritional assessment, SH had an interesting feature named nutrient balance score. During the day, it showed this score (0-100) based on the nutritional value of the recorded daily food intake. It was not clear if this was calculated from the macronutrient distribution only or micronutrients and other possible variables. Similarly, CC had a grade (eg, A-, D+, and F) for the nutritional analysis and highlighted with colors (ie, green, yellow, and red) if the nutrients were within the recommended threshold.

The apps also had output features related to PA and phenotype (see Table 5). Weight progress, shown in graphs, was found in all the apps. Five apps presented the body mass index (BMI) calculation. *Forums and blogs* were found in seven apps and used frequently for sharing recipes and tips about weight loss and diets. Most of the possibilities for *social media sharing* (eg, Facebook) were related to weight loss achievements. MFP allowed users to connect with their Facebook friends who were also using MFP, after requesting their permission.

In addition, this review identified the existence of *private social media*, defined as having a feature for “following” other users, adding them “as a buddy” or supporting them. This feature was considered the distinction between forums/blogs and private social media. FS provided an innovative feature for sharing the results with nutritionists and other health professionals, so that they could follow the monitoring online. NC and FS had a feature for exporting recorded data in a comma separated value (CSV) format. They did not export GPS data, but the results could be used for general data analysis or experiments.

As mentioned, MDC is not a food diary. It has a clear motivational focus using virtual rewards via the Healthy Habits (HH) points, which can be obtained by drinking more water, eating vegetables, or parking the car far away from one’s destination.

## Discussion

### Nutrition Assessment

The most popular dietary intake apps available in November 2015 used prospective nutrition assessments. The focus of the food diaries was on the balance between the food intake and energy expenditure, with personalized recommendation of diet plans not featuring in these apps. The four generic diet plans were based on a number of inputs required from the user—weight, height, gender, and age—without subsequent dietary intake assessment. The feature for saving favorite foods and meals is an effective time-saving feature, mainly for those who consume the same food items frequently. Three apps allowed the user to set a date for reaching a target weight, but only NC limited the weight loss rate.

There is a general focus on weight loss and calorie counting, with the majority of apps containing either *calorie* or *weight* in the title. It is important to note that nutrition assessment should not be related only to weight loss to target obesity, although this might be one of the main motivations for using nutrition-related apps. Ideal weights are not suggested to the users, but are sometimes required as inputs. The target date for reaching a specific weight is also entered by the user. However, if used without professional recommendation, this may mislead the users to begin unhealthy diets or trigger an eating disorder [11,12]. Although integration of food diaries and some types of PA monitoring have been successful, personalized nutrition advice is limited. The innovative feature of sharing results with health professionals might be a possible strategy for achieving part of this goal.

A quantitative approach is the usual strategy used by apps to balance the energy content of diets with energy expenditure. Data from the diet diary is used as the estimated energy intake and the basal metabolic rate, and the energy expended through physical activities as the energy expenditure. However, this method does not take into account the quality of foods consumed. For instance, the distribution of food groups, as recommended by some public health organizations, is not considered [13]. The score feature proposed by SH to assess the nutritional quality of the dietary intake might be an alternative to address this need. The textual feedback provided by MFP related to micronutrients and food grade mentioned in the CC nutrition facts might help users to gain some knowledge related to nutrients. The portion sizes are selected based only on text. Although the serving sizes can be useful in this situation, the apps do not present photos or icons for assisting the user to choose the most accurate portion size. Personalized advice based on health conditions or specific groups, such as vegetarians and vegans, was not available in the apps assessed.

All the apps collecting dietary intake used the same nutrition assessment method (ie, food diary record). However, there are alternative methods that are less time-consuming, such as the 24-hour recall method [14] and the FFQ [15,16], which have also been validated in Web-based formats.

### Technologies

Within the apps offering food diaries, aspects of PA monitoring were available via the use of GPS or wearables. These features allow users to monitor their outdoor activities (eg, walking and running) and the use of application programming interfaces (APIs) plays an important role in these integrations because they are created to facilitate the communication with other external apps. In general, the wearable devices collect data and save them in their own systems and allow third-party apps, such as the nutrition-related apps, to import that data via APIs. In addition, indoor activities can be logged by selecting the type of activity and duration. Using the same strategy, LS and MFP provided the possibility to import weight measurements from Withings body scales (Withings Inc, Cambridge, MA), which can measure weight, BMI, and heart rate and send this information via Wi-Fi to the Internet [17].

Emerging technologies, such as image recognition and natural language processing, are not present in the most popular nutrition apps. The combination of these technologies could simplify the food and portion selection processes. Image recognition seems to be promising for recognizing food items and estimating their portion sizes [18] and natural language processing could be used to transcribe spoken dietary records [19]. In academia, some studies using apps take advantage of specific hardware, such as laser beams attached to mobile phones [18], in order to increase the accuracy of the portion size estimation.

There is room for improvement in terms of connecting users and health professionals, in that the process of making diet recommendations could include more input from trained professionals. An automated system that offers personalized nutrition advice was proposed and developed by the Food4Me study, based on a decision tree created by nutritionists and dietitians [20]. A diet information system that connects dietitians and the public was proposed by Ravana et al in order to take advantage of artificial intelligence for proposing diet planning to the public [21]. In this context, artificial intelligence is used in an attempt to solve the diet planning challenge, so that the system can learn from past experiences (ie, similar scenarios). In theory, the combination of big data analytics and artificial intelligence would create a decision engine able to propose personalized online intervention [22-24]. A similar challenge is under investigation by IBM in a project named *cognitive cooking*, using these technologies to propose recipes to users [25]. These technologies were not featured in the apps assessed. This specific analysis could be a topic for future work in both academia and industry.

### Limitations

We acknowledge that the Google Play Store and the iTunes App Store have different app-ranking systems and market share. Hence, using the lowest number of reviews for the included Google Play Store apps as a threshold for including apps from the iTunes App Store may not reflect the number of downloads from the iTunes App Store. It is difficult to directly compare app popularity between the two stores, as the number of downloads from the iTunes App Store is not publically available. As the Google Play Store does not provide the exact number of installs, it is possible that some apps in the range *500,000-1m* could have approached 1 million installs. The criteria used to select the apps were based on the number of installs and reviews. Using these variables alone, it was not possible to identify the frequency and duration of use of these apps. This information would be valuable to measure the real engagement of the users and determine if they would accept the burden of text searching and barcode scanning for a prolonged period. It would also be interesting to assess the percentage of users that upgraded to the premium versions of the apps. Since it is not possible to measure the upgrades, the premium versions were not considered popular and their extra functionalities were not included in this review. A similar limitation occurred with the WW app, which requires a subscription [9]. Since the functionalities of these apps change rapidly, it is recommended that a similar assessment be conducted in the future. Although it is likely that these apps are also available and popular in other English-speaking countries, such as the United States and Canada, these results are limited to a UK perspective. A review of popular apps in different countries and languages could reveal other important features and interesting cultural differences.

### Comparison With Prior Work

Chen et al have recently published research assessing the most popular mobile phone apps for weight loss used in Australia [26]. They have developed a method for quantifying the quality of the apps and also assess the utilization of behavior change techniques (BCTs). However, given that a different methodology for defining the most popular apps was used in this study, and that the apps published in the online stores are distinct by country, only six out the 13 apps assessed in our study were alike. Some investigators have also conducted analyses of commercial nutrition-related apps in terms of content and health behavior theories [8,27,28]. This research complements and extends this prior work by providing a detailed analysis of the features offered by individual apps and also by analyzing what emerging technologies have been applied by them.

### Conclusions

A total of 13 apps that had at least 1 million installs were identified. Nine of the apps collected dietary intake, all using the same assessment method (ie, food diary record). Food selection was accomplished via text search and barcode scanning. Portion size selection was conducted by selecting text, and not by images or icons. Image recognition, natural language processing, and artificial intelligence did not feature in the apps. There is significant opportunity for improvement in terms of personalized nutrition, which could include individualized feedback, diet plans, or nutrition education.

## Abbreviations

API: application programming interface
BCT: behavior change technique
BMI: body mass index
CC: Calorie Count
CNPq: National Council of Technological and Scientific Development
CSV: comma separated value
DA: Diet Assistant
DP: Diet Point
EWL: Effective Weight Loss
FFQ: food frequency questionnaire
FS: FatSecret
GPS: global positioning system
HH: Healthy Habits
LI: Lose it!
LS: Lifesum
LW: Lose Weight Without Dieting
MDC: My Diet Coach
MDD: My Diet Diary
MFP: MyFitnessPal
N/A: not applicable
NC: Noom Coach
PA: physical activity
PC: personal computer
SH: S Health
WW: Weight Watchers
